# Assessing disparities in colorectal cancer mortality by socioeconomic status using new tools: health disparities calculator and socioeconomic quintiles

**DOI:** 10.1007/s10552-016-0842-2

**Published:** 2017-01-12

**Authors:** Nancy Breen, Denise Riedel Lewis, James Todd Gibson, Mandi Yu, Sam Harper

**Affiliations:** 1 Division of Cancer Control and Population Sciences, National Cancer Institute, 6707 Democracy Blvd, Suite 800 MSC 5465, Bethesda, MD 20892-5465 USA; 2 Office of Science Policy, Planning, Analysis and Reporting, National Institute on Minority Health and Health Disparities, Bethesda, MD USA; 3 Information Management Services Incorporated, Beltsville, MD USA; 4 McGill University, Montreal, Quebec Canada

**Keywords:** Disparities, Colorectal Cancer Mortality, HD*Calc, SES Quintiles, Concentration Index, Inequality Aversion Parameter

## Abstract

**Purpose:**

Colorectal cancer mortality rates dropped by half in the past three decades, but these gains were accompanied by striking differences in colorectal cancer mortality by socioeconomic status (SES). Our research objective is to examine disparities in colorectal cancer mortality by SES, using a scientifically rigorous and reproducible approach with publicly available online tools, HD*Calc and NCI SES Quintiles.

**Methods:**

All reported colorectal cancer deaths in the United States from 1980 to 2010 were categorized into NCI SES quintiles and assessed at the county level. Joinpoint was used to test for significant changes in trends. Absolute and relative concentration indices (CI) were computed with HD*Calc to graph change in disparity over time.

**Results:**

Disparities by SES significantly declined until 1993–1995, and then increased until 2010, due to a mortality drop in populations living in high SES areas that exceeded the mortality drop in lower SES areas. HD*Calc results were consistent for both absolute and relative concentration indices. Inequality aversion parameter weights of 2, 4, 6 and 8 were compared to explore how much colorectal cancer mortality was concentrated in the poorest quintile compared to the richest quintile. Weights larger than 4 did not increase the slope of the disparities trend.

**Conclusions:**

There is consistent evidence for a significant crossover in colorectal cancer disparity from 1980 to 2010. Trends in disparity can be accurately and readily summarized using the HD*Calc tool. The disparity trend, combined with published information on the timing of screening and treatment uptake, is concordant with the idea that introduction of medical screening and treatment leads to lower uptake in lower compared to higher SES populations and that differential uptake yields disparity in population mortality.

## Introduction

Colon and rectum cancer (hereafter colorectal cancer) is the second most common cause of cancer death in the United States [1]. Survival rates are much higher for early than for late stage disease [2]. Between 1975 and 2010, mortality rates from colorectal cancer in the United States for white males dropped from 35 to less than 20 per 100,000 population [3]. This striking drop in mortality over just three decades represents an astonishing accomplishment. In this study, we evaluate whether the reduction in colorectal cancer death rates was equally distributed throughout the population.

Summary measures combine information on mortality or other health outcomes to represent the distribution of population health in a single numerical index. To explore the social distribution of the decline in mortality over time, we use new tools developed at the National Cancer Institute (NCI) at the National Institutes of Health: the Health Disparities Calculator (HD*Calc) and the NCI Socioeconomic Status (SES) index. HD*Calc addresses the recommendation of an NCI monograph that multiple summary measures should be used to accurately evaluate trends in disparities [4]. The recommendation to use multiple measures resulted after examination of the performance and suitability of 23 summary measures of disparity. NCI developed the Health Disparities Calculator (HD*Calc) to create efficiencies in computations and graphing. HD*Calc is an online tool that calculates and graphs 11 summary disparity measures previously published in peer-reviewed journals [5]. The HD*Calc measures were selected from among those reviewed in the NCI monograph for their reliability and versatility.

Many dimensions of health disparity exist in the United States. Important differences in health outcome in populations’ signal disparity, and it is important to recognize the impact that social determinants have on health outcomes. Race, ethnicity, sex, sexual identity, age, disability, socioeconomic status, and geographic location all contribute to the ability to achieve good health and describe groups that have been historically subjected to discrimination or other social injustices [6]. Other studies have shown disparities in colorectal cancer mortality by race/ethnicity [7, 8]. We analyze disparities using HD*Calc by area socioeconomic status by linking mortality data to recently developed NCI SES quintiles.

The NCI SES quintiles are characterized by a standard set of factors. A standard set of factors reduces measurement error and improves precision over time. The quintiles are linked at the smallest ecological unit available (which, for mortality, is Census tract), quintiles are calculated for each year using the same factors, and changes in trends in quintiles are assessed for significance. The NCI SES quintiles, based on Census data that can be linked to individual data at the county or tract level, provide a consistent measure of SES [9]. SES is a key health determinant, and the previous analyses have shown large differences in cancer outcomes associated with SES over time [10–13].

In this study, we evaluate disparities in colorectal cancer mortality by SES using a set of summary indices, one relative and one absolute. We examine age-adjusted mortality by county SES from 1980 to 2010 for the United States. While annual rates show a mortality decline for all groups and an apparent crossover of mortality rates among SES groups, we cannot draw scientific conclusions without estimating measures of disparity and assessing their precision. Thus, we use HD*Calc and the NCI SES quintiles [2] to address two research questions. First, how large was the disparity in colorectal cancer mortality rates by SES group? Second, did SES disparities change direction over time? The indices we use are sensitive to the direction of the SES gradient and include an inequality aversion parameter which can be adjusted to specify how much weight to give to each socioeconomic group.

## Materials and methods

### Data

Colorectal cancer mortality data collected by the National Vital Statistics System and accessed through the NCI’s SEER program and Census data for the United States from 1980 to 2010 were combined at the county level; county is the smallest unit for which mortality data are released. Colorectal cancer mortality by SES was calculated for each NCI SES quintile for each year under study. Factor analyses were used to derive the NCI SES index scores from seven county attributes: educational attainment of persons aged 25 and over, median household income, median gross rent, median value (dollars) for owner-occupied housing units, percent of persons under 150% poverty level among population, percent unemployed among the civilian population aged 16 and over, and percent working class among the civilian employed population aged 16 years and over. These attributes were normalized using the rank transformation. The first factor that explained more than 80% of common variance was extracted for the index. SES quintiles were then formed from the index with equal populations within each quintile for each year.

Specifically, data from the 1980, 1990, and 2000 decennial Census long form surveys and the American Community Survey 5-year estimates (2005–2009, 2006–2010, and 2007–2011) were used to construct the indices. For Census years, we used the Census data. For intercensual years (1981–1989, 1991–1999, and 2001–2006), linear interpolated SES scores from the two adjacent Census years were used. For the years that coincide with the middle years of the ACS 5-year estimates (2007, 2007, and 2008), the ACS data were used. For 2010, linear extrapolated scores from the two previous sets of 5-year estimates of the ACS were used.

Numerators are derived from all reported deaths in the United States with colorectal cancer identified as the underlying cause. Colorectal cancer mortality data are maintained by the National Center for Health Statistics and disseminated through the Surveillance Epidemiology and End Results program [14]. Cause of Death Recodes [15] is based on the International Classification of Diseases version 10 (ICD-10) for year of death from 1999 to 2010 (ICD-10: C18–20, C26) and version 9 (ICD-9) for year of death from 1980 to 1998 (ICD-9: 153, 154.0–154.1, 159). For each year and county, colorectal cancer deaths were categorized into NCI SES quintiles with the first quintile being the lowest SES and the fifth quintile being the highest.

Denominator data from the bridged single race vintage 2012 population estimates [16] were grouped into NCI SES quintiles. We calculated age-standardized rates for all colorectal cancer deaths by dividing the numerators for each gender-specific quintile by its corresponding population estimate grouped by quintile and gender.

### Analysis

We used the recommended sequence of steps to assess health disparities from the NCI Monograph [4]. The first step is to examine the underlying “raw” data, in this case mortality by subgroup. Age-adjusted mortality rates were calculated using SEER*Stat [17]. Significance of mortality trends from 1980 to 2010 was calculated using Joinpoint Regression Program 4.3 [18, 19]. The Joinpoint regression tests for statistically significant changes over time in the magnitude and direction of the change of the outcome (e.g., mortality rate and size of health disparities). Joinpoint breaks a time series into connected straight line segments by estimating the numbers and locations of join points. The change rate in the outcome is considered constant between two adjacent points, which is also known as the trend for this segment. Two types of measures are commonly used to measure the trend. For outcomes that are often assumed to be distributed linearly on a log scale, for example, mortality rates, the annual percent change (APC) is used. For outcomes that do not require log-transformations, such as the health disparities measures that we examine in this study, a regression slope with interpretations that are similar to the one produced by simple linear regressions is used. In all Joinpoint regression analyses, the minimum and maximum numbers of join points were set to be zero and five, respectively, over the 1980–2012 period. Observations are assumed to be independent. Permutation tests were used to select the final model.

Using HD*Calc, we examined absolute and relative colorectal cancer mortality disparities by area-SES quintiles. If the social group has a natural ordering, such as SES, the NCI monograph recommends using the Slope Index of Inequality (SII) or the Absolute Concentration Index (ACI) to measure absolute health disparity, and the Relative Index of Inequality (RII) or the Relative Concentration Index (RCI) to measure relative disparity. The Concentration index, whether measured on the absolute or relative scale, places an additional weight on the health of disadvantaged groups relative to advantaged groups and includes an inequality aversion parameter which can be adjusted [4]. For all measures, a value of zero means no disparities (except for the Kunst–Mackenback RII for which a value of 1 means no disparities), a positive value indicates a disparity in mortality in favor of low SES, and a negative value indicates a disparity in mortality in favor of high SES. The standard inequality aversion parameter, which equals 2, weights the health of the poorest individual by 2 and, thereafter, declines to 0 with increasing SES [20]. The aversion parameter can be used to specify how much weight is assigned to different parts of the socioeconomic distribution. The Indices of Inequality and the Concentration Indices are mathematically similar, so there is no reason to compute both indices. Rather, different inequality aversion parameters for the ACI and the RCI were compared to observe how disparity levels and trends would change as a result.

Another step concerns relative and absolute measurement. Relative disparities are compared to a reference group and computed as a ratio. Absolute disparities are the difference between actual numbers. Importantly, these are different conceptual ways to measure disparities [21, 22]. Reporting just one can be misleading, and it is not uncommon that using different measures may lead to different conclusions regarding the direction of disparity trends [4]. In short, scientific accuracy requires measuring both relative and absolute concepts before drawing conclusions [23, 24]. Finally, the World Health Organization’s Commission on Social Determinants of Health, recommends reporting both absolute and relative measures for properly tracking health inequalities [25].

## Results

Table 1 shows deaths from colorectal cancer for each SES quintile by gender in the United States from 1980 to 2010. The table suggests an association between SES and death from colorectal cancer.

**Table 1:** Death count, population, and age-adjusted mortality rate by county socioeconomic quintile and gender, US Mortality Data, 1980–2010

SES quintile	Male			Female		
	Death (*n*)	Population (*n*)	Mortality rate^a^	Death (*n*)	Population (*n*)	Mortality rate
SES 1 (low)	107,529	185,120,920	56.50	85,133	211,349,031	37.70
SES 2	101,790	178,931,705	56.35	80,572	204,583,695	37.30
SES 3	94,881	166,659,249	57.75	75,191	190,281,024	38.08
SES 4	90,447	168,916,368	55.06	71,586	190,651,488	36.61
SES 5 (high)	86,476	170,295,271	54.77	67,750	188,101,664	36.52

^a^Mortality rates shown are per 100,000 person years

**Fig. 1:**
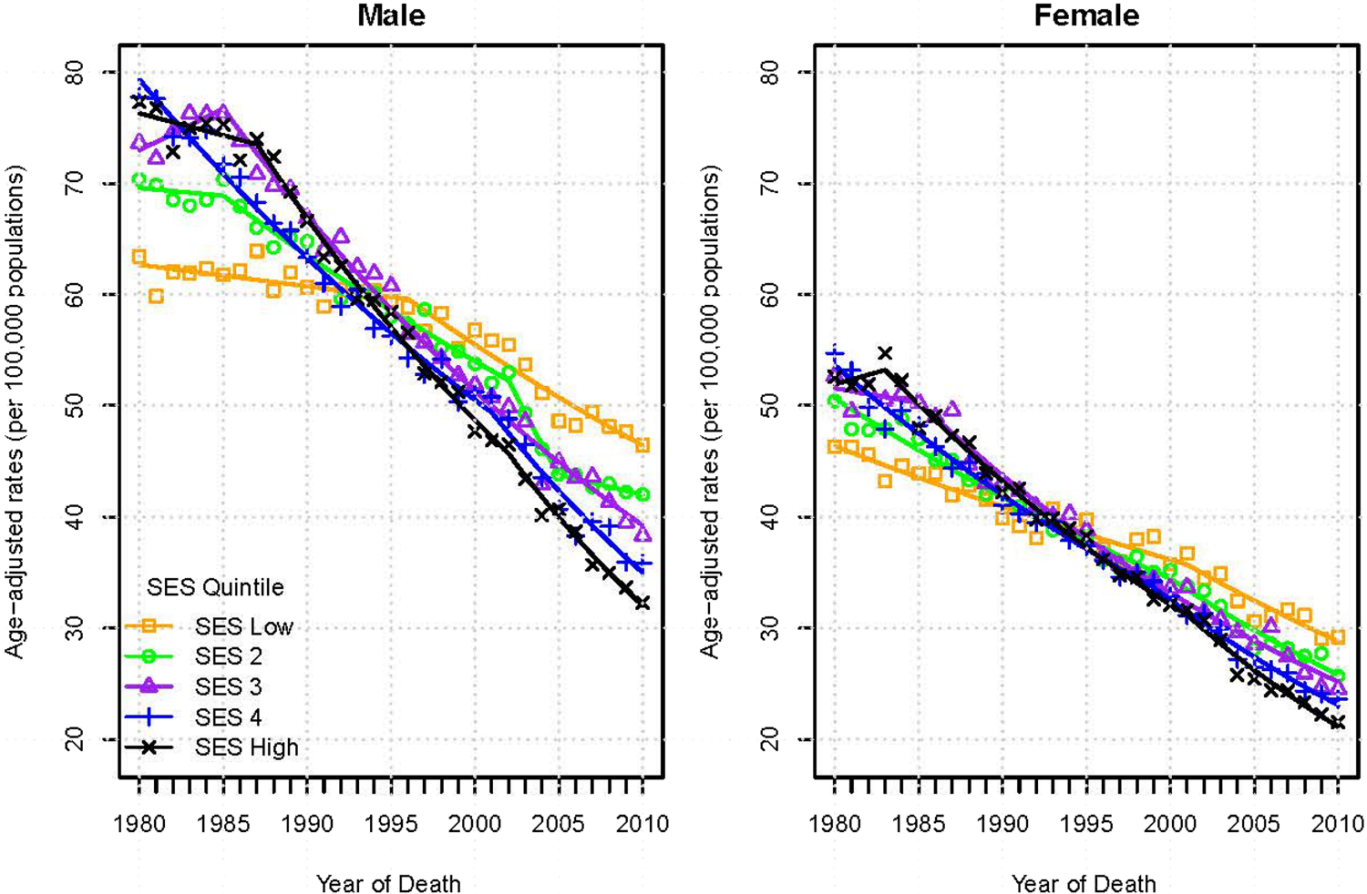
Joinpoint analysis of US Colorectal Cancer Mortality. Rates by SES quintile and gender, 1980–2010

Figure 1 presents the Joinpoint trend analysis of colorectal cancer mortality rates by gender and SES quintile over time using annual age-adjusted rates for the United States. In 1980, higher SES is associated with a larger share of deaths, while at the end of the period, higher SES is associated with a smaller percentage of deaths. This association is exacerbated for males compared to females.

Average percent change was statistically significant for most trends, as Table 2 shows. In 1980, colorectal cancer mortality rates were highest in the highest area-SES quintile, 80/100,000 and approximately 60/100,000 for men in the lowest quintile. By 2010, mortality rates for all men had dropped. Rates for men living in the higher SES areas dropped the most to about 32/100,000 (APC = −4.4, significant); rates for low SES men dropped to approximately 46/100,000 (APC = −1.8, significant). This striking crossover in mortality rates occurred around 1993.

**Table 2:** Joinpoint trend results for the US Colorectal Cancer Mortality Rates by SES (SES) quintile and gender, 1980–2010

SES quintile	Male					Female				
	No. of trends	Trend	Start year	End year	APC	No. of trends	Trend	start year	End year	APC
SES 1 (low)	2	1	1980	1996	−0.3*	2	1	1980	2001	−1.2*
		2	1996	2010	−1.8*		2	2001	2010	−2.4*
SES 2	4	1	1980	1985	−0.2	2	1	1980	2000	−1.9*
		2	1985	2002	−1.6*		2	2000	2010	−2.8*
		3	2002	2005	−5.7*		–	–	–	–
		4	2005	2010	−0.8		–	–	–	–
SES 3	2	1	1980	1985	1.0	2	1	1980	1985	−0.5
		2	1985	2010	−2.6*		2	1985	2010	−2.7*
SES 4	2	1	1980	2001	−2.2*	2	1	1980	1999	−2.4*
		2	2001	2010	−3.8*		2	1999	2010	−3.4*
SES 5 (high)	3	1	1980	1987	−0.5	3	1	1980	1983	0.8
		2	1987	2002	−3.1*		2	1983	2001	−2.9*
		3	2002	2010	−4.4*		3	2001	2010	−4.2*

*APC* annual percent change

*APC is significantly different from zero at the 0.05 level

**Fig. 2:**
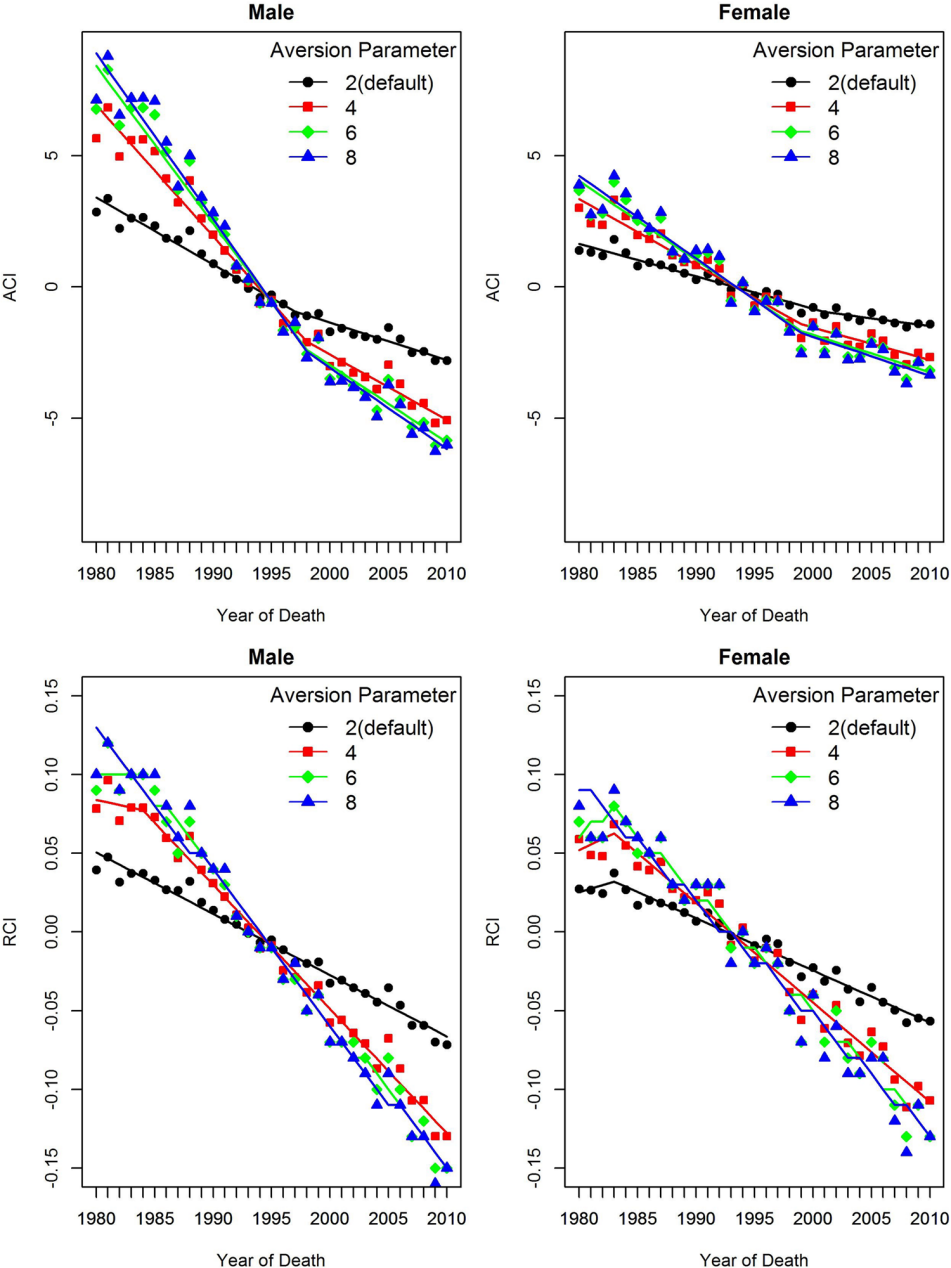
Extended concentration indices for socioeconomic disparities in colorectal cancer mortality rates, entire US, 1980–2010

Figure 2 shows results from HD*Calc. The Absolute Concentration Index (ACI) shows disparities in mortality by SES status started out positive, indicating mortality favored the low SES groups, and fell to zero around the mid-1990s, after which disparities became negative, indicating mortality favored the high SES group. Findings from the Relative Concentration Index (RCI) are consistent with the Absolute Concentration Index.

A key feature of the Extended Concentration Index is the inequality aversion parameter, which weights the health of the low-income quintile more than the high-income quintile. We used the inequality aversion parameter to explore the extent to which CRC mortality is more concentrated in the lowest income quintile compared to the highest quintile. Figure 2 presents findings for inequality aversion parameters set at 2 (the default), 4, 6, and 8. It is clear that larger inequality aversion parameters reflect greater concentrations of CRC mortality in the poorest quintile up to a weight of 4. Aversion parameters greater than 4 showed no additional effect.

## Discussion

### Summary of findings

Mortality from colorectal cancer dropped over the 30-year period from 1980 to 2010. At least for women, rates consistently dropped since the 1950s [26]. However, not all SES groups have benefitted equally. Joinpoint regression analyses found larger declines in the APC for individuals living in higher SES areas. The changes in trends were more gradual among women compared to men. Trends were consistent for absolute and relative summary measures.

The concentration indices confirmed reversal of the area-SES gradient over the past 30 years, and showed that presently disparities are favoring higher SES groups. Comparisons of some other cancer sites have shown relative and absolute disparities moved in opposite directions, but both relative and absolute disparity trends were in the same direction for both men and women for colorectal cancer mortality.

We also took advantage of the fact that the Extended Concentration Index has an inequality aversion parameter allowing users to specify how much to weight health in lower SES groups. We found larger disparities and stronger reversals of the SES gradient over time for both men and women with increasing weights up to 4 but little change for greater levels of inequality aversion.

### Advances in colorectal cancer screening and treatment

Medical procedures for colorectal cancer screening and treatment were developed starting in the 1980s. Screening techniques offer early detection, and treatment is more effective when cancer is detected early. Screening technology for colorectal cancer was not widely used before 2000 [27]. Surgery is the primary treatment modality for colorectal cancer and, from 1987 until 1995, adjuvant therapy which improved colorectal cancer outcomes, was developed and widely adopted [28]. Continued discovery and accelerated adoption of chemotherapy and radiation therapy between 1990 and 2010 contributed to still lower rates of mortality [29, 30]. However, these new procedures were not equally adopted by all SES groups [31–33].

Screening accounts for most of the early detection of colorectal cancer and, since 2000, colonoscopy has accounted for most of the increase in screening [34]. However, colonoscopy is expensive and higher SES adults are much more likely to report a recent colorectal cancer screening test [35]. In addition to early detection, colonoscopy identifies adenomas which are removed during the colonoscopy procedure, thus preventing colorectal cancer [36]. In this way, increased colonoscopy in a population leads to both a reduction in colorectal cancer incidence and a shift to earlier stage diagnosis. Earlier detection improved survival after surgery, and survival after surgery improved even more after 1990, as surgery was accompanied by adjuvant chemotherapy. Because colonoscopy use is concentrated in high SES populations, it seems plausible that differences in screening are implicated in the reductions in colorectal cancer mortality associated with higher SES status and with increasing SES disparities over time.

While expensive screening and treatment services are effective in reducing colorectal cancer mortality, their high cost makes it less likely that persons with low income or without health insurance will obtain them. As long as treatment and, especially, screening for CRC are costly to consumers in the United States, it seems likely that differential access will persist. The social gradient cannot be fully attributed to stage of disease, type of operation, specialization of surgery, or other clinical factors, because other studies have shown that the effect of SES on mortality persists after adjustment for these factors [37, 38]. It also is worth noting that another study documented faster declines in colorectal cancer mortality in higher SES areas since at least the 1950s, which would suggest that factors other than screening technology are also implicated in rising inequalities [11]. Nevertheless, unless the high cost of colorectal cancer screening declines, we are skeptical that historical stages 3 and 4, which suggest a lessening of inequality, will occur [12].

Another approach to understanding the association between colorectal cancer mortality and SES is to posit low SES as an etiological factor underlying disparities in colorectal cancer mortality. From this point of view, higher colorectal cancer mortality associated with low SES is preventable and can be addressed. However, addressing low SES directly is too broad to be efficacious in reducing colorectal cancer mortality rates. More promising are proven interventions that directly target barriers to screening and treatment.

### Contribution of this study

Our contribution to the literature is an accurate and reliable description of the trend in disparities in colorectal cancer mortality from 1980 to 2010. We confirmed disparities in colorectal cancer associated with SES between 1980 and 2010 using multiple summary disparity measures. Moreover, disparities are likely to continue to widen unless programs and policies are specifically directed to low SES populations to reverse the trend. Our findings on mortality are consistent with knowledge about screening and treatment, and with theories concerning why SES gaps could occur when overall mortality declines [12–15]. However, the previous studies could not be reproduced or compared, because different factors were included in the different SES quintiles. The NCI SES quintiles will be consistently measured using a standard set of factors.

The two free and open-source tools we used can improve estimates of disparities. The accuracy, reliability, and precision of measurement of summary trends in disparities depend on the quality and granularity of the data used for the calculations. The NCI SES quintiles are computed using a standard approach and provide more precise SES estimates over time, because each year is separately computed in a consistent way. The mortality database with linked NCI county SES quintiles is available upon request in SEER*Stat (http://seer.cancer.gov/resources/specialized.html). HD*Calc creates efficiencies in calculating tables and graphs for four absolute and seven relative indices of disparities. These tools can be used to update our findings as data become available.

### Limitations

Due to changes in the collection of Census data in 1980, comparable SES quintiles cannot be constructed for earlier years. SES quintiles are available at the Census tract level, a smaller geographic unit than county, but mortality data for colorectal cancer are not available at the Census tract level due to confidentiality concerns. We caution readers not to interpret county level SES status as a proxy for individual SES status. National time series data on utilization for the full range of medical treatment for colorectal cancer with geographic indicators are not available. Finally, studies of shifts in cancer stage by SES have not been conducted.

### Public health implications

Though the SES disparities that occurred with uptake of expensive new medical technologies may not be intended, it is not unexpected. Continued discovery and adoption of expensive screening and treatment for colorectal cancer [39, 40] suggest that this trend toward increasing disparities in colorectal cancer mortality may continue unless offsetting policies are put in place. New York [41] and Delaware [42] successfully established programs to promote equity in colorectal cancer screening. Delaware demonstrated that is it possible to achieve equity between African-Americans and whites in colon cancer screening, incidence, and mortality rates, even though it cannot be ascertained whether the program was responsible for the reduction in racial disparities. However, it does seem clear that concerted and dedicated public health efforts across the cancer control continuum are needed to avoid disparities emerging alongside medical improvements [43]. To avoid medical interventions becoming causes of disparities in colorectal cancer mortality, colorectal cancer screening and treatment need to be equally accessible to all SES groups. This means developing and funding programs that specifically target groups that may not know about or are unable to afford CRC screening and treatment. Shifting from screening with expensive colonoscopy to screening with effective and low-cost stool tests, such as the Fecal Immunochemical Test (FIT) or the stool DNA test, would make CRC screening more accessible [44]; however, currently, many providers charge co-pays for follow-up colonoscopy and this is a barrier to colorectal cancer screening [45]. Some resistance to high prices for cancer drugs is occurring along with recognition that the high cost of cancer treatment is leading to bankruptcies [46, 47].

Unless corrective measures are taken to promote and pay for routine screening, follow-up as needed, and timely affordable treatment in lower SES populations, medical service uptake for colorectal cancer will continue to be higher among higher SES patients and disparities will continue to widen. The example of colorectal cancer is especially striking, because, before medical interventions, populations in lower SES areas experienced lower mortality. Our evidence shows a crossover in colorectal cancer mortality by SES that is consistent with the introduction of new technologies and adds plausibility to the impact of SES: as new technology became available, screening, earlier stage diagnosis, and timely effective treatment for colorectal cancer were adopted earlier and used at higher rates in higher SES populations. For lower SES populations to equally benefit from screening and treatment, programs and policies would need to be implemented that would bring screening, diagnostic and treatment services to low SES populations.

To see results, programs and policies need to be accurately monitored over an extended period. Delaware mobilized its health care community toward the goal of eliminating racial health disparities in colorectal cancer. Disparities in screening, incidence, advanced stage of disease, and mortality between Whites and African-Americans were eliminated between 2001 and 2009. Whether the intervention was fully responsible for the elimination of racial disparities in colorectal cancer screening and mortality is unclear, because there was no control group. Future work should focus on better study designs to understand the mechanisms through which inequalities in colorectal cancer mortality are generated and sustained. Observational studies of colorectal cancer stage at diagnosis by SES to assess whether shifts in cancer stage by SES have occurred are important, and would be facilitated by the NCI SES Quintiles.

## References

[1] Howlader N, Noone AM, Krapcho M, Garshell J, Miller D, Altekruse SF, Kosary CL, Yu M, Ruhl J, Tatalovich Z, Mariotto A, Lewis DR, Chen HS, Feuer EJ, Cronin KA (eds) (2014) SEER cancer statistics review, 1975–2010. Chapter 1, Table 1.27. http://seer.cancer.gov/archive/csr/1975_2010/

[2] Howlader N, Noone AM, Krapcho M, Garshell J, Miller D, Altekruse SF, Kosary CL, Yu M, Ruhl J, Tatalovich Z, Mariotto A, Lewis DR, Chen HS, Feuer EJ, Cronin KA (eds) (2014) SEER cancer statistics review, 1975–2012. http://seer.cancer.gov/csr/1975_2012/

[3] Howlader N, Noone AM, Krapcho M, Garshell J, Miller D, Altekruse SF, Kosary CL, Yu M, Ruhl J, Tatalovich Z, Mariotto A, Lewis DR, Chen HS, Feuer EJ, Cronin KA (eds) (2015). SEER cancer statistics review, 1975–2012. Colon and rectum—tables and figures. http://seer.cancer.gov/csr/1975_2012/results_merged/sect_06_colon_rectum.pdf

[4] Harper S, Lynch J (2005). Methods for measuring cancer disparities: a review using data relevant to Healthy People 2010 cancer-related objectives. NCI Cancer Surveillance Monograph Series, Number 6.

[5] Breen N, Scott S, Percy-Laurry A, Lewis D, Glasgow R (2014). Health disparities calculator: a methodologically rigorous tool for analyzing inequalities in population health. Am J Public Health.

[6] Promotion OoDPaH (2010) Healthy People 2020. Healthy People. https://www.healthypeople.gov/2020/about/foundation-health-measures/Disparities

[7] Soneji S, Iyer SS, Armstrong K, Asch DA (2010). Racial disparities in stage-specific colorectal cancer mortality: 1960–2005. Am J Public Health.

[8] Gorey KM, Haji-Jama S, Bartfay E, Luginaah IN, Wright FC, Kanjeekal SM (2014). Lack of access to chemotherapy for colon cancer: multiplicative disadvantage of being extremely poor, inadequately insured and African American. BMC Health Serv Res.

[9] Yu M, Tatalovich Z, Gibson J, Cronin K (2014). Using a composite index of socioeconomic status to investigate health disparities while protecting the confidentiality of cancer registry data. Cancer Causes Control.

[10] Singh GK, Miller BA, Hankey BF (2002). Changing area socioeconomic patterns in US cancer mortality, 1950–1998: Part II—Lung and colorectal cancers. J Natl Cancer Inst.

[11] Harper S, Lynch J (2007). Selected comparisons of measures of health disparities: a review using databases relevant to Healthy People 2010 cancer-related objectives. NCI Cancer Surveillance Monograph Series, Number 7.

[12] Wang A, Clouston SA, Rubin MS, Colen CG, Link BG (2012). Fundamental causes of colorectal cancer mortality: the implications of informational diffusion. Milbank Q.

[13] Saldana-Ruiz N, Clouston SA, Rubin MS, Colen CG, Link BG (2013). Fundamental causes of colorectal cancer mortality in the United States: understanding the importance of socioeconomic status in creating inequality in mortality. Am J Public Health.

[14] NCHS (2013) Surveillance, Epidemiology, and End Results (SEER) Program (www.seer.cancer.gov). SEER*Stat Database: Mortality—Cancer, Total U.S. (1980-2010). National Cancer Institute, DCCPS, Surveillance Research Program, Surveillance Systems Branch. www.cdc.gov/nchs

[15] National Cancer Institute (2004) SEER cause of death recode 1969+. http://seer.cancer.gov/codrecode/1969+_d09172004/. Accessed 17 Sep 2004

[16] Centers for Disease Control and Prevention. National vital statistics system. US census populations with bridged race categories. http://www.cdc.gov/nchs/nvss/bridged_race.htm

[17] National Cancer Institute (2016) SEER*Stat Software. Version 8.3.2—April 14, 2016. https://seer.cancer.gov/seerstat/

[18] Kim HJ, Fay MP, Feuer EJ, Midthune DN (2000). Permutation tests for joinpoint regression with applications to cancer rates. Stat Med.

[19] Joinpoint Trend Analysis Software. Version 4.3. https://surveillance.cancer.gov/joinpoint/

[20] Wagstaff A (2002). Inequality aversion, health inequalities and health achievement. J Health Econ.

[21] Ravallion M (2016). The economics of poverty: history, measurement, and policy.

[22] Sen A (1983). Poor, relatively speaking. Oxford Econ Pap.

[23] King NB, Harper S, Young ME. (2012) Use of relative and absolute effect measures in reporting health inequalities: structured review. BMJ Br Med J 345 10.1136/bmj.e5774 PMC3432634 22945952

[24] Mackenbach JP, Kunst AE (1997). Measuring the magnitude of socio-economic inequalities in health: an overview of available measures illustrated with two examples from Europe. Soc Sci Med (1982).

[25] Kelly MP, Bonnefoy J. (2007) The social determinants of health developing an evidence base for political action: NHS

[26] Siegel R, DeSantis C, Jemal A (2014). Colorectal cancer statistics, 2014. CA Cancer J Clin.

[27] Breen N, Meissner HI (2005). Toward a system of cancer screening in the United States: trends and opportunities. Annu Rev Public Health.

[28] Potosky AL, Harlan LC, Kaplan RS, Johnson KA, Lynch CF (2002). Age, sex, and racial differences in the use of standard adjuvant therapy for colorectal cancer. J Clin Oncol Off J Am Soc Clin Oncol.

[29] Edwards BK, Ward E, Kohler BA, Eheman C, Zauber AG, Anderson RN, Jemal A, Schymura MJ, Lansdorp-Vogelaar I, Seeff LC, van Ballegooijen M, Goede SL, Ries LA (2010). Annual report to the nation on the status of cancer, 1975–2006, featuring colorectal cancer trends and impact of interventions (risk factors, screening, and treatment) to reduce future rates. Cancer.

[30] Murphy CC, Harlan LC, Lund JL, Lynch CF, Geiger AM (2015) Patterns of colorectal cancer care in the United States: 1990–2010. J Natl Cancer Inst 107(10) 10.1093/jnci/djv198 PMC4840367 26206950

[31] Aarts MJ, Lemmens VE, Louwman MW, Kunst AE, Coebergh JW (2010). Socioeconomic status and changing inequalities in colorectal cancer? A review of the associations with risk, treatment and outcome. Eur J Cancer (Oxford, England: 1990).

[32] Gorey KM, Luginaah IN, Bartfay E, Zou G, Haji-Jama S, Holowaty EJ, Hamm C, Kanjeekal SM, Wright FC, Balagurusamy MK, Richter NL (2013). Better colon cancer care for extremely poor Canadian women compared with American women. Health Soc Work.

[33] Chawla N, Butler EN, Lund J, Warren JL, Harlan LC, Yabroff KR (2013) Patterns of colorectal cancer care in Europe, Australia, and New Zealand. J Natl Cancer Inst Monogr 46:36–61 10.1093/jncimonographs/lgt009 PMC3888187 23962509

[34] Seeff L, Nadel MR, Klabunde CN, Thompson T, Shapiro J, Vernon SW, Coates R (2004). Patterns and predictors of colorectal cancer test use in the adult US population. Cancer.

[35] Klabunde CN, Cronin KA, Breen N, Waldron WR, Ambs AH, Nadel MR (2011). Trends in colorectal cancer test use among vulnerable populations in the United States. Cancer epidemiology, biomarkers and prevention: a publication of the American Association for Cancer Research, cosponsored by the American Society of Preventive. Int Soc Cell.

[36] Byers T, Levin B, Rothenberger D, Dodd GD, Smith RA (1997). American Cancer Society guidelines for screening and surveillance for early detection of colorectal polyps and cancer: update 1997. American Cancer Society Detection and Treatment Advisory Group on Colorectal Cancer. CA Cancer J Clin.

[37] Frederiksen BL, Osler M, Harling H, Ladelund S, Jørgensen T (2009). Do patient characteristics, disease, or treatment explain social inequality in survival from colorectal cancer?. Soc Sci Med.

[38] Frederiksen BL, Osler M, Harling H, Ladelund S, Jorgensen T (2009). The impact of socioeconomic factors on 30-day mortality following elective colorectal cancer surgery: a nationwide study. Eur J Cancer (Oxford, England: 1990).

[39] Schrag D (2004). The price tag on progress–chemotherapy for colorectal cancer. N Engl J Med.

[40] Ferro SA, Myer BS, Wolff DA, Poniewierski MS, Culakova E, Cosler LE, Scarpace SL, Khorana AA, Lyman GH (2008). Variation in the cost of medications for the treatment of colorectal cancer. Am J Manag Care.

[41] Neugut AI, Lebwohl B (2009). Screening for colorectal cancer: the glass is half full. Am J Public Health.

[42] Grubbs SS, Polite BN, Carney J, Bowser W, Rogers J, Katurakes N, Hess P, Paskett ED (2013). Eliminating racial disparities in colorectal cancer in the real world: it took a village. J Clin Oncol Off J Am Soc Clin Oncol.

[43] Cipriano TM, Polite BN (2013) Achieving health equity in colorectal cancer: a call to action. American Society of Clinical Oncology educational book/ASCO. American Society of Clinical Oncology. Meeting, pp 169–173 10.14694/EdBook_AM.2013.33.169 23714491

[44] American Cancer Society (2015) What’s new in colon cancer testing? http://www.cancer.org/cancer/news/features/understanding-tests-that-screen-for-colon-cancer

[45] Green BB, Coronado GD, Devoe JE, Allison J (2014). Navigating the murky waters of colorectal cancer screening and health reform. Am J Public Health.

[46] Pollack A Sanofi halves price of cancer drug Zaltrap after Sloan-Kettering rejection. The New York Times

[47] de Souza JA, Conti RM (2016) Mitigating financial toxicity among us patients with cancer. JAMA Oncol 10.1001/jamaoncol.2016.4850 27893021

